# Last glacial temperature reconstructions using coupled isotopic analyses of fossil snails and stalagmites from archaeological caves in Okinawa, Japan

**DOI:** 10.1038/s41598-021-01484-z

**Published:** 2021-11-09

**Authors:** Ryuji Asami, Rikuto Hondo, Ryu Uemura, Masaki Fujita, Shinji Yamasaki, Chuan-Chou Shen, Chung-Che Wu, Xiuyang Jiang, Hideko Takayanagi, Ryuichi Shinjo, Akihiro Kano, Yasufumi Iryu

**Affiliations:** 1 Institute of Geology and Paleontology, Graduate School of Science, Tohoku University, Aobayama, Sendai 980-8578 Japan; 2 Department of Earth and Environmental Sciences, Graduate School of Environmental Studies, Nagoya University, Furo-cho, Chikusa-ku, Nagoya, 464-8601 Japan; 3 Department of Anthropology, National Museum of Nature and Science, Tsukuba, Ibaraki 305-0005 Japan; 4 Okinawa Prefectural Museum & Art Museum, Okinawa, 900-0006 Japan; 5 High-Precision Mass Spectrometry and Environment Change Laboratory (HISPEC), Department of Geosciences, National Taiwan University, Taipei, 10617 Taiwan, ROC; 6 Research Center for Future Earth, National Taiwan University, Taipei, 10617 Taiwan, ROC; 7 Laboratory of Inorganic Chemistry, Department of Chemistry and Applied Biosciences, ETH Zurich, 8093 Zurich, Switzerland; 8 Key Laboratory of Humid Subtropical Eco-Geographical Processes, College of Geography Science, Ministry of Education, Fujian Normal University, Fuzhou, 350007 China; 9 Research Institute for Humanity and Nature (RIHN), Motoyama 457-4, Kamigamo, Kita-ku, Kyoto, 603-8047 Japan; 10 Department of Earth Science, Faculty of Science, University of the Ryukyus, 1 Senbaru, Nishihara, Okinawa 903-0213 Japan; 11 Department of Earth and Planetary Science, Faculty of Science, The University of Tokyo, 7-3-1 Hongo, Tokyo, 113-0033 Japan

**Keywords:** Climate sciences, Palaeoclimate

## Abstract

We applied a new geoarchaeological method with two carbonate archives, which are fossil snails from Sakitari Cave and stalagmites from Gyokusen Cave, on Okinawa Island, Japan, to reconstruct surface air temperature changes over the northwestern Pacific since the last glacial period. Oxygen isotope ratios (δ^18^O) of modern and fossil freshwater snail shells were determined to infer seasonal temperature variations. The observational and analytical data confirm that δ^18^O values of fluid inclusion waters in the stalagmite can be regarded as those of spring waters at the sites where snails lived. Our results indicate that the annual mean, summer, and winter air temperatures were lower by 6–7 °C at ca. 23 thousand years ago (ka) and 4–5 °C at ca. 16–13 ka than those of the present day. Our reconstruction implies that surface air cooling was possibly two times greater than that of seawater around the Ryukyu Islands during the Last Glacial Maximum, which potentially enhanced the development of the East Asian summer monsoon during the last deglaciation. Considering the potential uncertainties in the temperature estimations, the climatic interpretations of this study are not necessarily definitive due to the limited number of samples. Nevertheless, our new geoarchaeological approach using coupled δ^18^O determinations of fossil snails and stalagmite fluid inclusion waters will be useful for reconstructing snapshots of seasonally resolved time series of air temperatures during the Quaternary.

The Last Glacial Maximum (LGM; ca. 27,000–19,000 years ago^1^) is a well-studied paleoclimatic and paleoceanographic period in Earth’s history, and past glacial climates have been compared with present-day and Holocene warm climates. The dynamics of the East Asian monsoon (EAM), which has been a major component of Earth’s climate system throughout the late Quaternary, has been investigated using proxy-based reconstructions and climate simulation studies^2,3^. Recently, paleoclimate records from stalagmites^2,4^ and trees^5,6^ in East Asian continental regions and planktonic foraminifers^7,8^ and corals^9,10^ in the northwest subtropical and temperate Pacific have been used to delineate the history of the EAM. The former two and latter two archives document hydrological variations in the atmosphere and seawater temperature variations, respectively. Although the EAM is the result of thermal differences between the land and oceans, little is known about past surface air temperatures over the Northwest Pacific, especially during the LGM when global sea level was > 110 m lower than today^11^.

Seawater temperatures can be reliably reconstructed using geochemical proxies such as alkenones^12^, Mg/Ca ratios of foraminifers^13^, and Sr/Ca ratios of corals^14^ and bivalves^15^. Oxygen isotope values (δ^18^O) of marine biogenic carbonates can also be used as a paleo-thermometer after correcting for seawater δ^18^O variations associated with global ice volume^16^. Nevertheless, proxy records for atmospheric temperature are rare, except for ice core data from high-latitude areas^17^. A few studies have shown that δ^18^O values of aragonitic shells of shallow marine^18^ and freshwater^19^ snails reflect the temperature and δ^18^O values of waters in which they lived. However, past terrestrial water δ^18^O values cannot be directly determined. Recently, a new approach for determining δ^18^O values of very small amounts of fluid inclusion waters in stalagmites has been developed^20–22^, which can be used for reconstructing meteoric water δ^18^O values, such as for rainfall, spring waters, and river waters.

Quaternary reef deposits are widely distributed in the southern part of Okinawa Island, southwestern Japan^23^, and it is known that the island contains numerous limestone caves and speleothems. Recently, many ancient remains and fossils, such as human and mammalian bones, shells, and crustaceans, have been excavated from archaeological cave sites on the island^24,25^. In this study, we applied a new approach involving coupled δ^18^O analyses of fossil freshwater snail shells and fluid inclusion waters in stalagmites, in order to reconstruct paleo-air temperatures. We present the first seasonally-resolved time series of air temperature in the northwestern Pacific region for periods during the LGM and last deglaciation. Given that the East China Sea Shelf was extensively exposed due to the global sea level fall during the LGM^26^, the distance between the Eurasian continent and the Ryukyu Islands was much shorter than that today. Therefore, the maritime influence on the climate of Okinawa might have been reduced during the LGM. Our data allow a direct comparison of the surface air temperatures with seawater temperature records in the northwestern Pacific, and reveal differences in the behavior of the atmosphere and ocean in this region during the late Quaternary.

## Study site and samples

Okinawa Island (26°–27° N, 127.5°–128.5° E) is located in the subtropical climate of the Kuroshio Current region in the Ryukyu Islands, northwestern Pacific Ocean (Supplementary Fig. S1a). The regional climatology is affected by the EAM, typhoons, and the Kuroshio Current. At present (1991–2020 AD), air temperatures vary from 15.1 ± 0.8 °C in January or February to 27.4 ± 0.6 °C in July or August, with an annual average of 21.5 ± 0.3 °C (1σ) (data from the Japan Meteorological Agency [JMA] meteorological station “Itokazu Station” in Nanjo City) (Fig. S1a). The average annual rainfall amount is 2029 ± 524 mm (1σ), with two major rainy periods in the “Baiu” season in May–June and typhoon season in August–September. In winter, East Asian climate is influenced by the northwesterly and northerly prevailing, dry and cold winds from continental China and the Siberian High. The EAM affects the air and sea surface temperatures on and around Okinawa Island at present, and during the Holocene^10^, because of its location near the southern limit of the winter monsoon region. In summer, the island has a warm and wet climate with frequent typhoons, which are generally linked to the EAM intensity. The summer EAM brings southeasterly and southerly prevailing winds with a relatively high moisture content from the warm oceanic waters in the region of the Subtropical Ogasawara High and low-latitude oceans. Rainfall isotope data and an isotope-incorporated atmospheric general circulation model have revealed that seasonal differences in moisture sources on Okinawa Island generate marked variations in rainfall δ^18^O and hydrogen isotope (δD) values, with higher and lower values in winter and summer, respectively^27^.

Fossil shells of a freshwater snail *Semisulcospira* sp. were excavated in Sakitari Cave. This is a limestone cave at an archaeological site, which is located ~ 40 m above sea level on Okinawa Island, Japan (26°08′ N, 127°44′ E) (Fig. S1a, b). The cave is partly collapsed and dry. Previous archaeological studies have demonstrated that humans probably began to use the cave at ca. 31–29 thousand years ago (ka, cal. BP = before 1950 AD), based on radiocarbon (^14^C) ages of charcoal and freshwater snail shells collected from near human remains^24,25^. These studies also proposed that numerous marine and freshwater shells excavated from the cave sediments were collected from outside the cave as food and then discarded. The ^14^C dating results for 42 samples are highly consistent with the stratigraphy, indicating continuous deposition during 36.5–13.0 ka without any erosion or hiatuses. The cave sediments are well protected beneath a Holocene flowstone layer (11.0–2.8 ka) (Fig. S1c). In this study, fossil freshwater snail samples were recovered from Layers I (16.1–13.4 ka) and II-2 (23.1–22.5 ka) of the well-stratified sediments in the cave^24^ (Supplementary Table S1, Fig. S1c). The fossil samples were not directly dated, but their ages correspond to the respective ^14^C ages of the sediment layers. Living samples of the same species were collected at two nearby sites, Kakinohana and Kadeshi springs, in 2018 (Table S1, Figs. S1, S2). A calcite stalagmite (GYKN-2), which is 40 cm in length and 10 cm in diameter, was also collected from Gyokusen Cave. This is a limestone cave connected to Sakitari Cave in the same cavern system (Fig. S1a). The environment in Gyokusen Cave is characterized by a high relative humidity of > 95% and air CO_2_ of > 1000 ppm throughout the year. Two deposition segments of the stalagmite at 34.5–84.0 mm and 160–232 mm (from the tip) were U–Th dated and yielded age windows of 13.0–15.8 ka and 22.2–23.4 ka, respectively (see the “Methods” section for details) (Table S2, Fig. S3b). For oxygen and hydrogen isotope analyses of the stalagmite calcite and fluid inclusion water, six and four sub-samples were taken from the upper (13.8–15.2 ka) and lower (22.4–23.4 ka) parts of the stalagmite, respectively (Table S3, Fig. S3b). These periods correspond to the ages of Layer I and Layer II-2 in Sakitari Cave, respectively. Flowing-water samples in Gyokusen Cave were collected about every two months in 2010–2012. Spring water samples at Kakinohana and Kadeshi springs were collected in March and September 2019 and December 2020 to evaluate the water isotopic compositions around the study sites. The water temperatures and pH values were measured during field investigations in 2010–2020.

## Results and discussion

### δ^18^O values in a water system

**Figure 1:**
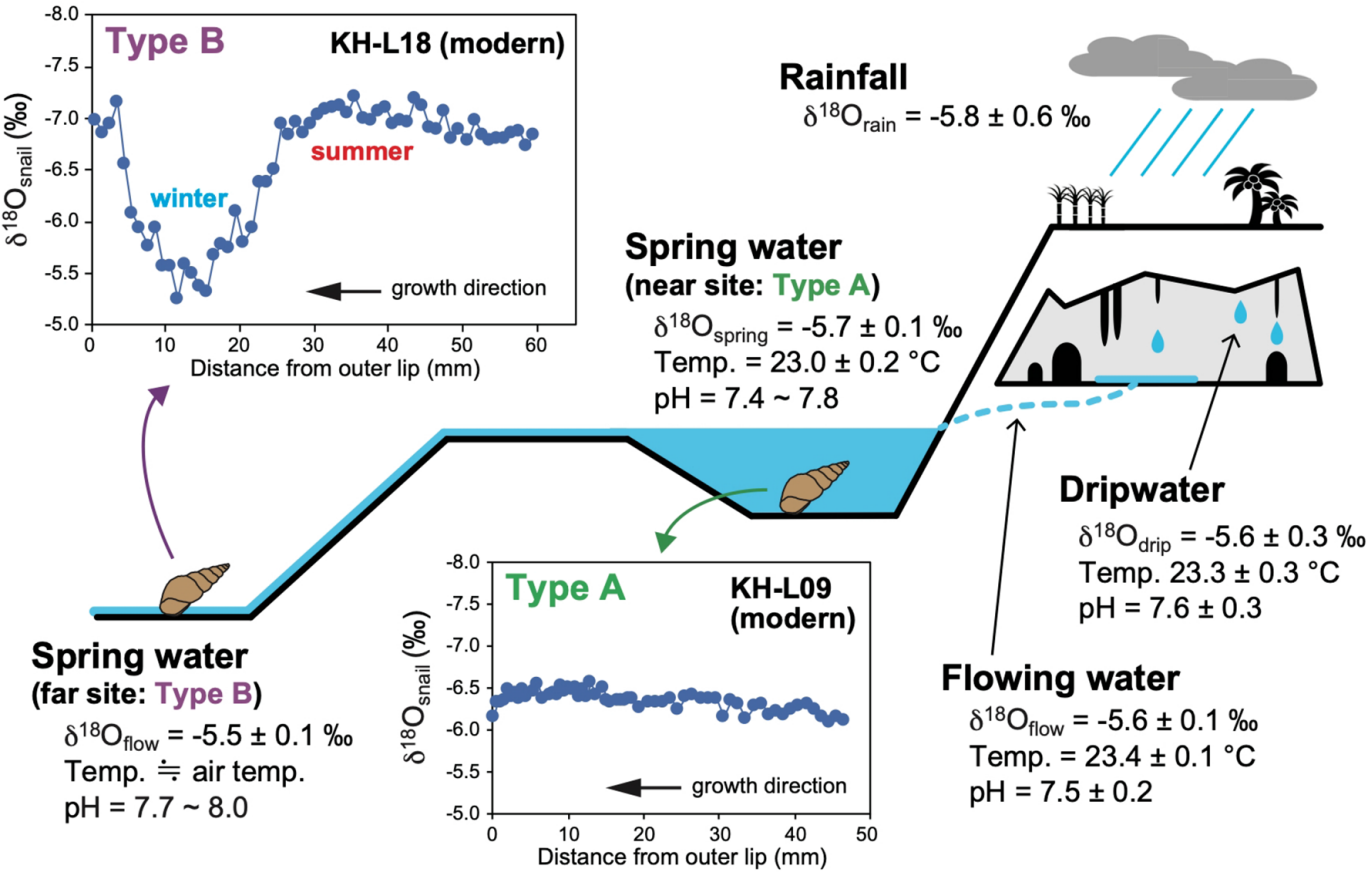
Schematic illustration showing a water system comprising rainfall^27^, drip water^21^, and flowing water (this study) in Gyokusen Cave, and spring water (this study) at Kakinohana near the cave. At a nearby site (Type A) and a far site (Type B) from the spring, representative δ^18^O profiles were generated from modern freshwater snails living completely in water and very shallow water, respectively.

Mean δ^18^O values of drip waters (δ^18^O_drip_)^21^ and flowing water (δ^18^O_flow_) (this study) in Gyokusen Cave for 2010–2012 were − 5.6 ± 0.3‰ and − 5.6 ± 0.1‰, respectively. These values are consistent with the weighted annual average rainfall δ^18^O (δ^18^O_rain_) value of − 5.8 ± 0.6‰ for 2009–2012^21,27^ (Fig. 1). The average spring water δ^18^O (δ^18^O_spring_) value is − 5.7 ± 0.1‰, which is identical to the values in the cave system. Furthermore, the temperature and pH of these waters are almost identical and lack seasonal variations (Fig. 1), implying that water evaporation and air ventilation hardly occur in Gyokusen Cave throughout the year. The analytical and observational data indicate that waters in the cave system have almost constant δ^18^O values throughout the year because of the long water retention time. δ^18^O values of fluid inclusion waters (δ^18^O_inclusion_) in two modern stalagmites, which grew in the cave over the past several decades, are almost similar to the δ^18^O_drip_ values^21^. These results show that δ^18^O_inclusion_ values of stalagmites in the cave can be regarded as an analogous to δ^18^O_spring_ values around the study area.

For δ^18^O_inclusion_ analysis, six and four sub-samples were taken from the upper (15.2–13.8 ka) and lower (23.4–22.4 ka) parts of the stalagmite, respectively (Fig. S3b). The ages of the fluid inclusion water samples were estimated based on linear interpolations between the stalagmite U–Th ages (Fig. S3). The arithmetic mean δ^18^O_inclusion_ values are − 4.9 ± 0.6‰ at 15.2–13.8 ka and − 4.9 ± 0.3‰ at 23.4–22.4 ka (Table S3), which are ~ 0.7‰ higher than the present-day δ^18^O_drip_ value of − 5.6‰. Similarly, the arithmetic mean δD values of the fluid inclusion waters (δD_inclusion_) are − 27.3 ± 3.3‰ at 15.2–13.8 ka and − 26.1 ± 2.9‰ at 23.4–22.4 ka (Table S3), which are ~ 6‰ higher than the present-day value of − 33‰. The δ^18^O difference is broadly consistent with the simulation results of the surface ocean around the Ryukyu Islands between the LGM and late Holocene^28,29^. Observational and model–experimental results have revealed that rainfall during winter has higher δ^18^O values by ~ 4‰ than in summer at present^27^. These lines of evidence indicate that δ^18^O_rain_ values were higher in Okinawa during the two selected periods of the last glaciation, because δ^18^O values of seawater (δ^18^O_sea_) were higher due to the ice volume effect and/or because the relative amount of winter to summer rainfall was higher than it is today. δ^18^O_inclusion_ and δD_inclusion_ values at 15.2–13.8 ka and 23.4–22.4 ka plot around the present-day meteoric water line (Fig. S4). The *d*-excess values of the fluid inclusion waters are 7.5–17.0‰ and 11.5–16.6‰, respectively, and do not differ greatly from the average value (12.2‰) of modern drip waters (Table S3). Consequently, these isotopic results imply that the cave water system and rainfall vapor source during the two studied periods in the last glaciation were broadly similar to those of today.

### Shell δ^18^O records of modern and fossil snails

Aragonitic shell δ^18^O values of modern freshwater snails (δ^18^O_snail_) can be categorized into two types in this study based on their habitat (Fig. 1). At a site near the spring (Type A) in the Kakinohana region, water temperatures have remained almost stable, with little seasonal variation (23.0 ± 0.1 °C for 2016–2017). The δ^18^O_snail_ value of a modern sample, living completely in water (Type A), had almost constant values of − 6.5 to − 6.0‰ (mean = − 6.3‰) (Figs. 1, S5). This is consistent with δ^18^O_snail_ records of modern samples, which have mean values of − 6.3‰ and − 6.1‰, with small variations of ~ 0.5‰ (Fig. S5), from nearly the same environment (≈ Type A) at Kadeshi Spring, that is ~ 6.5 km from the studied caves (Fig. S1). However, at a site farther from the spring (Type B) in the Kakinohana region, water temperatures exhibit significant seasonal variations associated with the air temperature, with average fluctuations of ~ 12 °C (data from the JMA meteorological station), because of the very shallow water depth of < 1 cm (Fig. 1). δ^18^O_snail_ values of modern samples exposed to the open air in the Type B setting exhibit seasonal variations ranging from − 7.4 to − 5.2‰, with means of − 6.5 to − 6.2‰ (Figs. 1, S5). Seasonal δ^18^O variations have also been observed in marine^18,30^ and freshwater^19^ gastropods, reflecting temperature variations during their growth. The line of monitoring results and modern δ^18^O_snail_ data suggests that fossil snails living in deep water such as Type A settings and in very shallow water such as Type B settings can be analyzed to reconstruct annual mean values in air temperature and both annual mean and seasonal variations, respectively.

**Figure 2:**
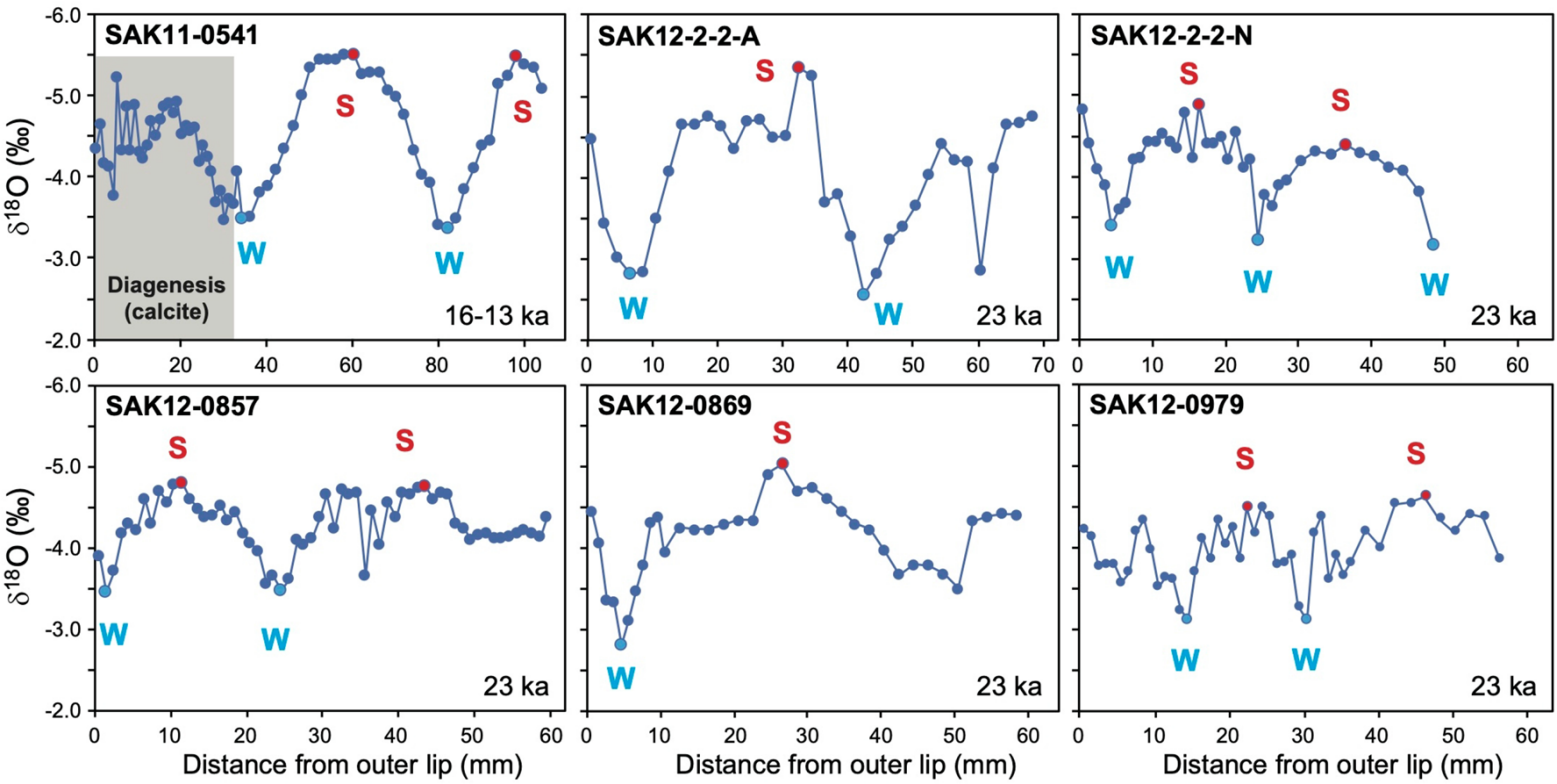
δ^18^O values of fossil freshwater snails showing seasonal temperature variations (S = summer; W = winter). The δ^18^O data for sample SAK11-0541 with calcite cement (shown in gray) were not used for the temperature reconstruction. Sample SAK11-0541 and the other samples were excavated from Layer I (16.1–13.4 ka) and Layer II-2 (23.1–22.5 ka) in Sakitari Cave, respectively. The lowest and highest values in a seasonal cycle were assigned to annual maximum (red circles) and minimum (blue circles) temperatures during summer and winter, respectively.

The δ^18^O_snail_ values of well-preserved fossil samples show seasonal variations over a period of a few years (Fig. 2). The δ^18^O_snail_ results indicate that the fossil freshwater snails analyzed in this study probably lived in very shallow water similar to a Type B setting (Fig. 1). A ca. 23 ka fossil snail sample containing diagenetic products (calcite) was not used for the reconstruction (Table S1). For the ca. 16–13 ka sample (SAK11-0541), the δ^18^O_snail_ record of the part of the shell portion unaffected by diagenetic alteration was used for the reconstruction (Fig. 2). δ^18^O_snail_ values of the ca. 23 ka samples vary from − 5.3 to − 4.4‰ in summer and − 3.5 to − 2.6‰ in winter, with annual averages of − 4.1 to − 3.8‰ (Fig. 2). The average summer and winter, and annual mean values of the ca. 16–13 ka snail were − 5.5‰, − 3.4‰, and − 4.5‰, respectively. The fossil samples have higher annual mean δ^18^O_snail_ values by > 1.9‰, as compared with the modern samples (Table 1), which cannot be solely explained by the 0.7‰ higher values of δ^18^O_inclusion_ (approx. − 4.9‰) relative to δ^18^O values of modern water (approx. − 5.6‰). This indicates that the climatic conditions in Okinawa were characterized by ^18^O-rich rainfall and lower temperatures during the LGM and last deglaciation as compared with today.

**Table 1:** Estimates of annual mean, summer, and winter temperatures at ca. 16–13 ka and ca. 23 ka.

Age	Sample ID	Shell δ^18^O (‰)			Water δ^18^O (Avg ± 1σ, ‰)	Δ*T* (°C, vs. modern)			Δ*T* (°C, vs. 1891–1950)					
		Annual mean	Summer	Winter		Annual mean	Summer	Winter	Annual mean	Summer	Winter	Average	Summer	Winter
												Stalagmite-water δ^18^O	Shell δ^18^O^24^	
Modern	KH-L09	− 6.35			− 5.59 ± 0.29									
	KH-L18	− 6.22	− 7.21 [*1*]	− 5.24 [*1*]										
	KH-L19	− 6.36	− 7.33 [*1*]	− 5.38 [*1*]										
	KH-L20	− 6.43	− 7.32 [*1*]	− 5.54 [*1*]										
	Avg ± 1σ	− 6.34 ± 0.09	− 7.29 ± 0.07	− 5.39 ± 0.15										
ca. 16–13 ka	SAK11-0541	− 4.47	− 5.48 [*2*]	− 3.44 [*2*]	− 4.90 ± 0.63	− 5.5	− 5.2	− 5.9	− 4.2 ± 3.3	− 3.9 ± 3.3	− 4.6 ± 3.3	− 6.6 ± 2.7	− 4.2 ± 3.3	− 7.3 ± 3.4
ca. 23 ka	SAK12-2-2-A	− 4.01	− 5.33 [*1*]	− 2.68 [*2*]	− 4.91 ± 0.28	− 7.8	− 6.0	− 9.5	− 6.6 ± 2.0	− 6.8 ± 2.4	− 6.3 ± 2.5	− 5.7 ± 2.0	− 6.2 ± 2.0	− 8.3 ± 2.1
	SAK12-2-2-N	− 3.96	− 4.64 [*2*]	− 3.28 [*3*]		− 8.0	− 9.2	− 6.7						
	SAK12-0857	− 4.12	− 4.77 [*2*]	− 3.48 [*2*]		− 7.2	− 8.6	− 5.8						
	SAK12-0869	− 3.94	− 5.04 [*1*]	− 2.83 [*1*]		− 8.1	− 7.3	− 8.8						
	SAK12-0979	− 3.84	− 4.57 [*2*]	− 3.12 [*2*]		− 8.5	− 9.6	− 7.4						

Italicized numbers within brackets denote the summer and winter shell δ^18^O data. Drip water and stalagmite inclusion water δ^18^O data represent spring water values. Temperature deviations were estimated from δ^18^O differences between the modern and fossil shells and waters, and from the coeval δ^18^O values of stalagmite calcite and fluid inclusion waters.

### Paleo-temperature calculations

The δ^18^O difference between aragonite and water is a function of temperature at the time of precipitation. We applied a temperature dependence of − 0.213‰/°C derived from a widely accepted equation^31^, which is in good agreement with previously published values of − 0.213‰/°C^32^ and − 0.217‰/°C^33^ for marine aragonite. To estimate relative surface air temperatures with respect to the present, the following equation was used:

1 $$ \Delta T = ((\delta^{\mathit{18}} O_{{{\text{fossil}}}} {-}\delta^{\mathit{18}} O_{{{\text{inclusion}}}} )- (\delta^{\mathit{18}} O_{{{\text{modern}}}} -\delta^{\mathit{18}} O_{{{\text{spring}}}} )) \times (-0.213)^{{-1}} $$

where Δ*T* represents the temperature difference between the past and present. *δ*^*18*^*O*_fossil_, *δ*^*18*^*O*_inclusion_, *δ*^*18*^*O*_modern_, and *δ*^*18*^*O*_spring_ are the oxygen isotope values of fossil snails, stalagmite fluid inclusion waters, modern snails, and modern spring waters, respectively. Based on the law of propagation of data error, the errors on the temperature reconstruction were estimated from the root-sum-square of the standard deviations of *δ*^*18*^*O*_fossil_, *δ*^*18*^*O*_inclusion_, *δ*^*18*^*O*_modern_, and *δ*^*18*^*O*_spring_ values.

**Figure 3:**
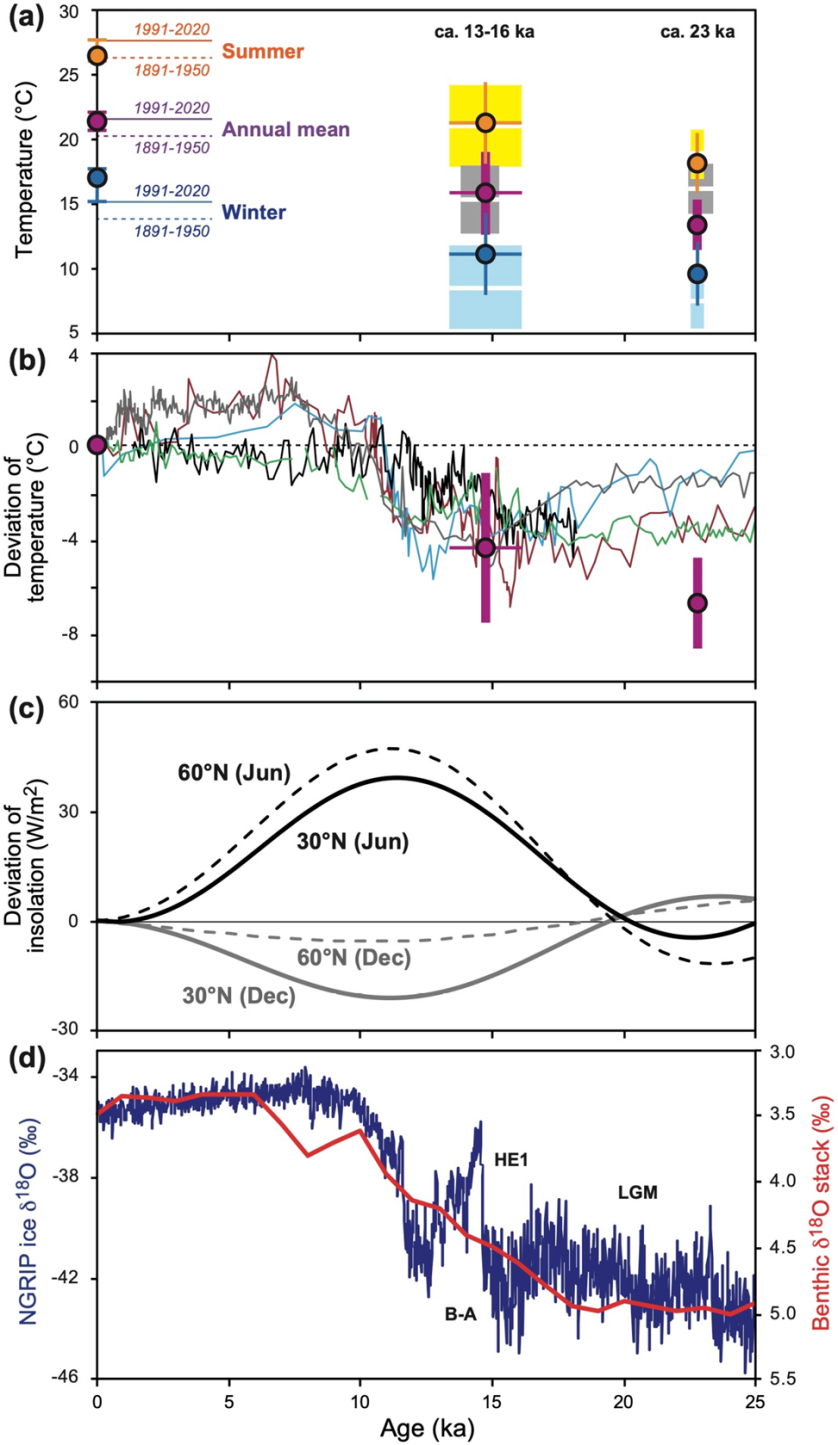
(**a**) Reconstructed annual mean (purple), summer (orange), and winter (blue) air temperatures (± 1σ) on Okinawa Island at 23.1–22.5 ka and 16.1–13.4 ka. Present-day average air temperatures around the study site were estimated from data from the Itokazu meteorological station, and are shown for 1891–1950 (dotted line) and 1991–2020 (solid line). Air temperatures reconstructed from a combination of coeval stalagmite calcite and fluid inclusion water δ^18^O data at 23.4–22.4 ka and 15.2–13.8 ka are shown in white (average) and gray (± 1σ). Reconstructed air temperatures from previously reported fossil snail δ^18^O data^24^ using our method are also shown (summer = white and yellow; winter = white and light blue). (**b**) A comparison of relative annual mean temperatures (purple; this study) with relative seawater temperature variations obtained from planktonic foraminifers and alkenones in deep-sea sediments in Okinawa Trough: black^7^ and green^43^ and off the eastern coast of Japan: light blue^41^, gray^42^, and brown^44^. The present-day value of zero is based on 1891–1950 for this study and the late Holocene for the others. (**c**) Deviations of June and December insolation at 30° N and 60° N relative to today^39^. (**d**) Greenland ice core δ^18^O record from the North Greenland Ice Core Project (NGRIP) (blue^37^) and benthic foraminifer δ^18^O stack record for the Pacific Ocean (red^38^). Probable timings of the late LGM, the Heinrich Event 1 (HE1), and Bølling–Allerød (B–A) are indicated. Paleoclimate data are available at the NOAA NCDC data archive (https://www.ncdc.noaa.gov/data-access/paleoclimatology-data).

Based on the observational and analytical data, the δ^18^O_inclusion_ values at 15.2–13.8 ka and 23.4–22.4 ka can be regarded as the δ^18^O values of spring waters where the fossil snails lived (Table 1, Fig. 1). Long-term meteorological monthly data from Naha Station (Fig. S1a) show that the annual mean, maximum (summer), and minimum (winter) air temperatures are 22.0 ± 0.4 °C, 28.0 ± 0.5 °C, and 15.5 ± 1.0 °C for 1891–1950, and 23.3 ± 0.4 °C, 29.2 ± 0.5 °C, and 16.9 ± 0.8 °C for 1991–2020, respectively. This clearly demonstrates that the climate of Okinawa has been approximately 1.3 °C warmer in 1991–2020 as compared with 1891–1950. Given that previously published relative seawater temperatures from planktonic foraminifers and alkenones in deep-sea sediments are compared with core-top data for the late Holocene, the years 1891–1950 should be used as the benchmark period for the estimation of relative temperature values in this study. As such, a correction of 1.3 °C was applied to our modern data (Table 1, Fig. 3).

The modern (Type B) and fossil δ^18^O_snail_ records typically exhibit one or two sine-like oscillations (Figs. 2, S5), which likely correspond to seasonal temperature variations of the ambient water during their growth. Similar δ^18^O_snail_ variations have been reported for many *Semisulcospira* sp. fossils (80%; 28 out of 35 samples) excavated from Sakitari Cave^24^. A monthly resolved time series of δ^18^O_snail_ values cannot be accurately established from the distance domain data, because little is known about seasonal and intra-seasonal variations in shell growth rate. Consequently, in this study, the lowest and highest δ^18^O_snail_ values in a seasonal cycle were taken to be the annual maximum and minimum temperatures during summer and winter, respectively (Figs. 2, S5). Given that shell growth rates of snails are uncertain and probably variable, the reconstructed mean temperature from all δ^18^O_snail_ values in a single shell does not necessarily reflect the actual annual mean value. In fact, the mean will be skewed towards the season with a higher growth rate, and thus higher sampling density. For modern monthly air temperature data from Itokazu Station, the annual mean value is equal to the intermediate value between the maximum and minimum monthly values, yielding an insignificant difference of + 0.2 ± 0.4 °C. Therefore, we used the intermediate values between the annual lowest and highest δ^18^O_snail_ values as the annual mean value (Table 1). However, for the sample KH-L09 at the Type A site, which does not show seasonal variations in δ^18^O_snail_, the mean of all values was taken to be the annual mean value.

Using the equation for inorganically precipitated aragonite δ^18^O values^31^, the annual average water temperatures estimated from the δ^18^O_modern_ and δ^18^O_spring_ values at the Type B site are in good agreement with the air temperatures recorded at Itokazu Station for 2017–2018 (i.e., whilst the snails were alive), yielding an insignificant difference of − 0.2 ± 0.6 °C. However, the summer and winter temperature estimates from the δ^18^O_modern_ and δ^18^O_spring_ values are 1.2 ± 0.3 °C lower and 1.9 ± 0.7 °C higher, respectively, than the recorded air temperatures. These data indicate that seasonal variations in water temperature at the Type B site are somewhat smaller than those of the air temperature. Therefore, the possible offsets of + 1.2 °C for summer and − 1.9 °C for winter were included in the temperature estimates for ca. 16–13 ka and ca. 23 ka (Fig. 3a), assuming that the fossil snails lived in a habitat similar to the modern snails. Although a potential vital effect on the biogenic carbonates cannot be excluded, it is assumed in this study that biologically derived errors in δ^18^O_snail_-based temperature estimations will be small, because the modern and fossil freshwater snails are the same species and similar in size (Table S1, Fig. S2). Given that seasonal growth cessation commonly occurs in freshwater mollusks in winter (e.g., < 8 °C^34^ and < 12 °C^35^), the potential errors on the winter temperature reconstructions during the last glacial may be larger than expected.

### Evaluation of uncertainties in the paleo-temperature reconstruction

δ^18^O_stalagmite_ values of four 23.4–22.4 ka and six 15.2–13.8 ka sub-samples are − 4.4 to − 3.8‰ and − 4.7 to − 3.3‰, respectively (Table S3). Based on the stalagmite calcite–water δ^18^O relationship^36^, paleo-temperatures were calculated from the coeval δ^18^O_stalagmite_ and δ^18^O_inclusion_ values. The calculated temperatures relative to today are − 7.5 to − 3.3 °C for the 23.4–22.4 ka sub-samples and − 9.4 to − 1.9 °C for the 15.2–13.8 ka sub-samples (Table S3). The calculated average temperatures were lower than today by 5.7 ± 2.0 °C (*n* = 6) at 23.4–22.4 ka and 6.6 ± 2.7 °C (*n* = 4) at 15.2–13.8 ka (Table 1, Fig. 3a). These are consistent with the annual average paleo-temperatures estimated from the δ^18^O_snail_ values, considering their errors. Furthermore, we applied our new approach to the previously reported lowest and highest δ^18^O_snail_ values of fossil samples from Sakitari Cave^24^, and obtained summer and winter paleo-temperatures that were lower than today by 6.2 ± 2.0 °C and 8.3 ± 2.1 °C at ca. 23 ka and by 4.2 ± 3.3 °C and 7.3 ± 3.4 °C at ca. 16–13 ka. These reconstructions agree with those from the δ^18^O_snail_ values of our samples (Table 1, Fig. 3a). These lines of evidence can demonstrate the robustness of our paleo-temperature reconstructions.

Uncertainties in the δ^18^O_snail_-based temperature reconstructions are potentially caused by: (i) analytical errors on the δ^18^O measurements; (ii) intraspecific (i.e., = inter-specimen) variations in the δ^18^O_snail_ values; (iii) age differences between the fluid inclusion waters and fossil snails; (iv) local differences in habitats between the modern and fossil snails. The root-mean-square of analytical errors on the *δ*^*18*^*O*_fossil_, *δ*^*18*^*O*_inclusion_, *δ*^*18*^*O*_modern_, and *δ*^*18*^*O*_spring_ measurements is ± 0.16‰, yielding an uncertainty of ± 0.75 °C for the paleo-temperature reconstruction due to (i). The standard deviations of the annual mean, summer, and winter δ^18^O_modern_ values result in temperature uncertainties of ± 0.40 °C (*n* = 4), ± 0.31 °C (*n* = 3), and ± 0.70 °C (*n* = 3), respectively, due to (ii). Given that the chronological data have ranges, robust temporal comparison of the fluid inclusion water and fossil snail data is not possible. In this study, the potential uncertainty due to (iii) is the range of ^14^C ages (16.1–13.4 ka and 23.1–22.5 ka; Fig. 3). It is uncertain whether the fossil snails lived in a hydrological environment exactly like the Type B site (Fig. 1), which potentially results in additional uncertainty on the summer and winter temperature reconstructions. Based on the δ^18^O_modern_ calibration, the uncertainty due to (iv) may reduce the seasonal variation of the reconstructed paleo-temperatures by ~ 3 °C, or more, in some cases. Further estimations from a larger number of fossil samples and culturing experiments of modern samples are required to reduce these uncertainties.

### LGM and last deglacial temperature reconstruction

Our results demonstrate that annual mean, summer, and winter air temperatures on Okinawa Island were 6.6 ± 2.0 °C, 6.8 ± 2.4 °C, and 6.3 ± 2.5 °C lower at ca. 23 ka, and 4.2 ± 3.3 °C, 3.9 ± 3.3 °C, and 4.6 ± 3.3 °C lower at ca. 16–13 ka than today (Table 1, Fig. 3a). These estimates are broadly supported by temperature reconstructions of a combination of coeval δ^18^O_stalagmite_ and δ^18^O_inclusion_ data, and previously reported δ^18^O_snail_ data^24^. These relative variations can be harmonized with the North Greenland Ice Core Project (NGRIP) ice core record^37^ and the benthic foraminifer δ^18^O stack for the Pacific Ocean^38^, which record Northern Hemisphere temperature variations and δ^18^O_sea_ variations since the last glacial (Fig. 3d). Although the uncertainty on our temperature estimates is > 1 °C, our results indicate that the air temperatures on Okinawa Island during the Bølling–Allerød (B–A) were higher by ~ 1.7 °C in winter and ~ 3 °C in summer than during the LGM. The greater increase in air temperature during summer relative to winter may be partly explained by the difference in North Hemisphere insolation changes during winter and summer. Insolation at 30° N and 60° N in June increased by ~ 35% and ~ 47% from ca. 23 ka to ca. 16–13 ka, which is different to the changes for December^39,40^ (Fig. 3c). However, this climatic interpretation should be considered preliminary, because only a 2-year-long time series at ca. 16–13 ka was extracted from a single fossil shell in this study. The meteorological data show that modern air temperatures vary by ~ 3 °C in summer and ~ 4 °C in winter. Although summer and winter temperature reconstructions using other δ^18^O_snail_ data^24^ are consistent with our results, analysis of more fossil shells and a culturing study of snail biomineralization are needed to reduce the uncertainties, and more specifically determine the temperature seasonality during the last deglaciation.

Our estimates of the annual mean temperature reveal an increase of ~ 2.4 °C from ca. 23 ka to ca. 16–13 ka for Okinawa Island (Fig. 3a,b). However, planktonic foraminifal Mg/Ca- and alkenone-derived seawater temperature records for the northwestern Pacific^7,41–44^ do not record such a large increase from the LGM to B–A (Fig. 3b). An alkenone record for the middle Okinawa Trough^43^ shows that the seawater temperature was ~ 3.5 °C and ~ 2.3 °C lower at ca. 23 ka and ca. 16–13 ka than the present, indicating slight warming of ~ 1 °C in the shallow waters around Okinawa Island. These results indicate that surface air warming was approximately two times larger than seawater warming throughout this period. Given the estimated errors, the stalagmite-derived temperatures in the cave do not significantly differ between the LGM and B–A (Fig. 3a). The fossil snail records imply that annual air temperatures for the selected time windows had larger variations or were cooler than those in the cave during the LGM. Considering the multiple uncertainties on the temperature estimates, this climatic interpretation is not necessarily definitive.

At ca. 16–13 ka, our estimate (approx. − 4.2 °C) of the relative air temperature as compared to today is 1–2 °C lower than the relative seawater temperature obtained from sediment records in the Ryukyu Islands and off the eastern coast of Japan (Fig. 3b). Sr/Ca data for a fossil Faviidae coral^45^, which commonly lives at 5–30 m water depth, showed that the annual mean sea surface temperature at ca. 16 ka was ~ 5 °C lower than the present, which is consistent with our results (Table 1). These estimates are lower by ~ 2 °C than seawater temperatures derived from Mg/Ca data of planktonic foraminifer *Globigerinoides ruber*, which commonly lives at water depths of < 100 m, in the Okinawa Trough^7^, northern East China Sea^8^, and northwestern Pacific Ocean^46^. This shows that temperature variations in the atmosphere and at the sea surface are faster and larger than for the deeper ocean. Furthermore, it is probable that cooling of the atmosphere as recorded in this study was much larger (> 3 °C) during the LGM (ca. 23 ka) than for seawater as reconstructed from alkenone records in the Okinawa Trough and off the eastern coast of Japan (Fig. 3b).

Okinawa Island had pollen assemblages that were dominated by coniferous trees such as *Pinus* and *Podocarpus* during the LGM (ca. 22 ka), indicating arid climate conditions at this time^47^. This finding is not in conflict with our estimates of higher δ^18^O_rain_ values and lower air temperatures at that time. The LGM annual mean air cooling of − 6.6 ± 2.0 °C reconstructed in this study is similar to the estimate of − 8.2 ± 2.4 °C (i.e., − 6.9 °C relative to pre-1950) from a combination of δ^18^O_stalagmite_ and δ^18^O_inclusion_ data from Okinawa Island at ca. 26 ka^21^. These data are consistent with a continental air temperature in East Asia of about − 8 °C relative to the present day, as inferred from a loess plateau^48^ and climate simulation results of − 4 to − 8 °C for Taiwan^49^ and the East China Sea region^50,51^. Recently, based on a paleo-thermometer using noble gases in groundwater, global land surface temperatures were estimated to be 5.8 ± 0.6 °C lower in low- to mid-latitude regions between 45° S and 35° N during the LGM as compared with today^52^. This global estimate appears to be consistent with our reconstructions, but does not account for local variations around our study site. One potential cause for local variation is that surface air cooling was much greater than that of seawater in the Ryukyu Islands, and is thought to have been related to geographic and oceanographic variations caused by global sea level change. Based on a sea level fall of > 110 m relative to today, which occurred during the LGM^11^, the East Asian land areas had expanded southeast. In addition, the eastern coast had extended ~ 150 km from Okinawa Island at that time (~ 650 km away at the present), although the Kuroshio Current flowed into Okinawa Trough with slightly reduced transportation^26^. Consequently, the last glacial air temperature on Okinawa Island is presumed to have been sensitive to continental climate change, especially winter EAM variations characterized by strong northerly to northwesterly, cold and dry winds.

A temperature reconstruction based on combining 956 geochemical sea surface temperature proxies with an isotope-enabled climate model ensemble using data assimilation indicated that global mean cooling of about − 6 °C occurred at the LGM^53^. To further evaluate regional differences in temperature between the land and ocean, reliable archives of air temperature are needed. However, there are few of these from oceanic areas. Given the coral-based evidence of interannual and decadal climate variations at the sea surface around the Ryukyu Islands and western tropical Pacific associated with the EAM, El Niño/Southern Oscillation, and Pacific Decadal Oscillation^10,54,55^, the snapshots of air temperature variations for selected time windows from this study do not necessarily show the representative mean values during the LGM and the B–A periods. To reduce the uncertainties on the δ^18^O_snail_-based paleo-temperatures, further investigations should be needed. However, we have presented a new approach for reconstructing annual mean, summer, and winter air temperatures using coupled δ^18^O determinations of fossil freshwater snails and fluid inclusion waters in stalagmites. Numerous carbonate islands in the tropical and sub-tropical Indo-Pacific region contain limestone caves that have been used to investigate the migration and culture of peoples in the late Pleistocene^24,25,56–58^, which would be amenable to our geoarchaeological and geochemical approaches for further temperature reconstructions.

## Methods

### Preservation tests

After removing organisms and soil, fossil and modern shells of the freshwater snail samples were washed using a brush and ultrasonically cleaned using ultrapure water. Selected fossil snail shell samples without traces of having been burnt (i.e., without charred and/or discolored shells) were used in this study (Fig. S2). To identify the presence or absence of calcite cements and diagenetic alteration, we conducted X-ray diffraction (XRD) analysis (X’Pert-MPD PW3050; Philips) and scanning electron microscopy (SEM) observations (3D VE-8800; Keyence) at Tohoku University, Japan, following procedures used in earlier studies^59,60^. Compared with the modern shells, the SEM observations confirmed that the fossils were well preserved and had experienced little diagenetic alteration or dissolution. XRD analysis showed that most fossils consisted of > 99.9% aragonite. Only two samples had very small amounts of calcite cement (< 3%; Table S1). Geochemical data for parts of shells with calcite cements were not used for climatic interpretations (Fig. 2).

### U–Th dating

The stalagmite GYKN-2 was cut along its growth axis, polished, and ultrasonically cleaned with ultrapure water. Uranium–thorium (U–Th) dating determination of two selected depth segments, corresponding to the coeval periods of the studied fossil snails (34.5–84.0 mm and 160–232 mm from the tip), was performed at the High-Precision Mass Spectrometry and Environment Change Laboratory (HISPEC), National Taiwan University, by using methods described earlier^61–63^ (Table S2, Fig. S3b). Isotopic measurements were conducted by using a multi-collector inductively coupled plasma mass spectrometer (NEPTUNE; Thermo Fisher Scientific). ^230^Th ages (thousand years ago, ka, relative to 1950 AD) were calculated with the determined U–Th isotopic compositions and contents, half-lives^64^, and an assumed ^238^U/^235^U atomic ratios of 137.818^65^. Uncertainties on the reported ages are given at 2σ level.

### Stable isotope analysis

Powdered sub-samples (~ 0.1 mg each) for geochemical profiling were taken every 1 mm along the growth direction of modern and fossil freshwater snail shells (Fig. S3a). Stable oxygen isotope ratios (δ^18^O_snail_) of aragonite shell samples were measured with a continuous flow isotope ratio mass spectrometer coupled to a Gasbench II and GC-PAL auto-sampler (Delta V Advantage; Thermo Fisher Scientific) at Tohoku University, Japan, following the methods described earlier^10^. For the oxygen and hydrogen isotope analyses of the stalagmite fluid inclusion waters (δ^18^O_inclusion_ and δD_inclusion_), six and four sub-samples were taken from the upper (15.2–13.8 ka) and lower (23.4–22.4 ka) parts of the stalagmite, respectively (Table S3, Fig. S3b). Sample preparation followed the methods described earlier^21,22^. In brief, to minimize the effects of the sample position relative to the growth axis on water contents, several wedge-shaped sub-samples (62–240 mg) were extracted from each part of the stalagmite. δ^18^O_inclusion_ and δD_inclusion_ values were measured by a cavity ring-down spectroscopy (CRDS L2130-i; Picarro) coupled to an extraction device at the University of the Ryukyus, Japan, following the methods reported earlier^21,22^. In summary, a stalagmite sub-sample was gently crushed under vacuum. The extracted water vapor was then trapped immediately and transferred to the CRDS analyzer for isotopic analysis. The entire system was heated to 105 °C. The isotope ratios of environmental water samples were measured simultaneously using a CRDS spectrometer with a vaporizer unit (L2130-i and V1120-i; Picarro, at the University of the Ryukyus, Japan, and 2120-i and A0211; Picarro, at Nagoya University, Japan). Powdered six sub-samples (15.2–13.8 ka) and four sub-samples (23.4–22.4 ka) of the stalagmite calcite used for the δ^18^O_inclusion_ measurements were analyzed for stable oxygen isotopes (δ^18^O_stalagmite_). Isotope ratios are reported in the conventional δ notation relative to Vienna Pee Dee Belemnite (VPDB) for carbonate and Vienna Standard Mean Ocean Water (VSMOW) for water. External precisions (1σ) are ± 0.05‰ for δ^18^O_snail_ and δ^18^O_stalagmite_, ± 0.3‰ for δ^18^O_inclusion_ and ± 1.6‰ for δD_inclusion_, and ± 0.08–0.17‰ for δ^18^O and ± 0.26–0.50‰ for δD for the environmental water samples. Data accuracy was evaluated based on replicate analyses of the standards GSJ/AIST JCp-1 aragonite, IAEA calcite standard CO-1, and SLAP water.

## Supplementary Information

Supplementary Information.

## Data Availability

All data are included in this published article and its Supplementary Information file.
