# Supplementary material for: Last glacial temperature reconstructions using coupled isotopic analyses of fossil snails and stalagmites from archaeological caves in Okinawa, Japan
Source: Sci Rep. 2021 Nov 9;11:21922. doi: 10.1038/s41598-021-01484-z (PMC8578419; doi:10.1038/s41598-021-01484-z)
Supplement: Supplementary file 1 — Supplementary Information. [file 41598_2021_1484_MOESM1_ESM.pdf]

Scientific Reports

Supplementary Information for

Last glacial temperature reconstructions using coupled isotopic analyses of fossil snails and stalagmites from archaeological caves in Okinawa, Japan

Ryuji Asami[1,*], Rikuto Hondo[1], Ryu Uemura[2], Masaki Fujita[3], Shinji Yamasaki[4], Chuan-Chou Shen[5,6], Chung-Che Wu[5,7], Xiuyang Jiang[8], Hideko Takayanagi[1], Ryuichi Shinjo[9,10], Akihiro Kano[11], Yasufumi Iryu[1]

[1] *Institute of Geology and Paleontology, Graduate School of Science, Tohoku University, Aobayama, Sendai 980-8578, Japan*

[2] *Department of Earth and Environmental Sciences, Graduate School of Environmental Studies, Nagoya University, Furo-cho, Chikusa-ku, Nagoya 464-8601, Japan*

[3] *Department of Anthropology, National Museum of Nature and Science, Ibaraki 305-0005, Japan*

[4] *Okinawa Prefectural Museum & Art Museum, Okinawa 900-0006, Japan*

[5] *High-Precision Mass Spectrometry and Environment Change Laboratory (HISPEC), Department of Geosciences, National Taiwan University, Taipei 10617, Taiwan, ROC*

[6] *Research Center for Future Earth, National Taiwan University, Taipei 10617, Taiwan, ROC*

[7] *Laboratory of Inorganic Chemistry, Department of Chemistry and Applied Biosciences, ETH Zurich, 8093 Zurich, Switzerland*

[8] *Key Laboratory of Humid Subtropical Eco-Geographical Processes, Ministry of Education, College of Geography Science, Fujian Normal University, Fuzhou 350007, China*

[9] *Research Institute for Humanity and Nature (RIHN), Motoyama 457-4, Kamigamo, Kita-ku, Kyoto 603-8047, Japan*

[10] *Department of Earth Science, Faculty of Science, University of the Ryukyus, 1 Senbaru, Nishihara, Okinawa 903-0213, Japan*

[11] *Department of Earth and Planetary Science, Faculty of Science, The University of Tokyo, 7-3-1 Hongo, Tokyo 113-0033, Japan*

* *Corresponding author: Ryuji Asami*
Phone number: +81-22-795-6616, Fax: +81-22-795-6634
Email: ryuji.asami.b5@tohoku.ac.jp

Contents of this file

Supplementary Tables S1 to S3
Supplementary Figures S1 to S5

Table S1. Details of the modern and fossil samples of the freshwater snail *Semisulcospira* sp.

Site[a]	Age	Sample ID	Size (mm)						XRD result	
			spire	body whorl	height	aperture	base	width	aragonite (%)	calcite (%)
Kakinohana Spring (A)	modern	KH-L09	8.4	14.4	22.9	9.6	6.9	10.2	100.0	0.0
Kakinohana Spring (B)	modern	KH-L18	11.1	15.9	27.1	10.6	7.8	11.7	100.0	0.0
		KH-L19	12.2	15.1	27.3	10.5	7.8	10.9	100.0	0.0
		KH-L20	12.1	15.5	27.6	10.3	7.6	10.3	100.0	0.0
Kadeshi Spring (A)	modern	KDS-L08	9.8	14.4	24.2	10.0	9.1	10.2	100.0	0.0
		KDS-L09	9.0	15.5	24.6	10.8	8.5	10.1	100.0	0.0
		KDS-L10	14.8	10.8	25.6	10.9	8.6	11.1	100.0	0.0
Sakitari Cave: Layer I (16.1–13.4 ka)	ca. 16-13 ka	SAK11-0541	8.3	16.4	24.7	11.1	9.1	11.3	97.3	2.7
Sakitari Cave: Layer II-2 (23.1–22.5 ka)	ca. 23 ka	SAK12-2-2-A	10.1	19.8	29.9	10.5	8.8	14.0	100.0	0.0
		SAK12-2-2-N	9.8	15.3	25.1	10.5	6.9	9.9	100.0	0.0
		SAK12-0857	13.8	18.5	32.3	12.6	9.7	11.9	100.0	0.0
		SAK12-0869	10.2	18.1	28.3	12.3	10.0	12.7	97.7	2.3
		SAK12-0979	8.5	17.5	26.0	12.6	9.9	12.5	100.0	0.0

[a] (A) a near site from spring with deep water depth (>10 cm); (B) a far site from spring with shallow water depth (<1 cm)

Table S2. Uranium–thorium (U–Th) isotopic compositions and ^{230}Th ages of sub-samples of the stalagmite GYKN-2

Horizon (mm)	Weight (g)	^{238}U (10^{-9} g/g)^{a}		^{232}Th (10^{-12} g/g)		$\delta^{234}U$ (measured)^{a}		[$^{230}Th/^{238}U$] (activity)^{c}		[$^{230}Th/^{232}Th$] (atomic x 10^{-3})^{d}		Age (ka, uncorrected)		Age (ka, corrected)^{c,e}		$\delta^{234}U_{initial}$ (corrected)^{b}	
* 34.5	0.1424	122.7	± 0.2	112	± 3	46	± 2	0.1184	± 0.0006	2.1	± 0.1	13.1	± 0.1	13.0	± 0.1	47	± 2
* 50	0.1445	82.60	± 0.09	2734	± 7	43	± 1	0.138	± 0.002	0.069	± 0.001	15.5	± 0.2	14.6	± 0.5	45	± 1
* 68	0.1397	62.0	± 0.1	18	± 3	43	± 3	0.1340	± 0.0008	7	± 1	15.0	± 0.1	14.9	± 0.1	45	± 3
* 84	0.1131	129.5	± 0.2	391	± 4	37	± 3	0.141	± 0.001	0.77	± 0.01	16.0	± 0.1	15.8	± 0.1	39	± 3
* 160	0.1631	108.9	± 0.2	1774	± 5	28	± 3	0.193	± 0.002	0.196	± 0.002	22.7	± 0.3	22.2	± 0.3	30	± 3
** 195	0.1185	77.5	± 0.1	168	± 4	28	± 2	0.198	± 0.001	1.51	± 0.04	23.4	± 0.1	23.2	± 0.1	30	± 2
** 232	0.1186	79.0	± 0.1	50	± 4	28	± 2	0.200	± 0.001	5.2	± 0.4	23.5	± 0.2	23.4	± 0.2	30	± 2

[*,**] Chemistry was performed on June 1st, 2012 (*) and on July 7th, 2015 (**) (Shen et al., 2003), and analyses on MC-ICP-MS (Shen et al., 2012). Analytical errors are 2σ of the mean.

[a] [^{238}U] = [^{235}U] X 137.818 (±0.65‰) (Hiess et al., 2012); $\delta^{234}U$ = ([$^{234}U/^{238}U$]$_{activity}$ - 1) X 1000.

[b] $\delta^{234}U_{initial}$ corrected was calculated based on ^{230}Th age (T), i.e., $\delta^{234}U_{initial} = \delta^{234}U_{measured} \times e^{\lambda_{234} * T}$, and T is corrected age.

[c] $[^{230}Th/^{238}U]_{activity} = 1 - e^{-\lambda_{230} T} + (\delta^{234}U_{measured}/1000)[\lambda_{230}/(\lambda_{230} - \lambda_{234})](1 - e^{-(\lambda_{230} - \lambda_{234}) T})$, where T is the age.

Decay constants are 9.1705×10^{-6} yr^{-1} for ^{230}Th, 2.8221×10^{-6} yr^{-1} for ^{234}U (Cheng et al., 2013), and 1.55125×10^{-10} yr^{-1} for ^{238}U (Jaffey et al., 1971) .

[d] The degree of detrital ^{230}Th contamination is indicated by the [$^{230}Th/^{232}Th$] atomic ratio instead of the activity ratio.

[e] Age, relative to AD 1950, corrections for samples were calculated using an estimated atomic $^{230}Th/^{232}Th$ ratio of 4 (± 2) X 10^{-6}.

Those are the values for a material at secular equilibrium, with the crustal $^{232}Th/^{238}U$ value of 3.8. The errors are arbitrarily assumed to be 50%.

Table S3. δ^{18}O and δD values of fluid inclusion waters and calcite in the stalagmite GYKN-2

Rference	Sample ID	Distance from top (mm)	Age (ka)	$CaCO_3$ Weight (mg)	H_2O (ppm)	δ^{18}O (‰)	Avg. ±1σ	δD (‰)	Avg. ±1σ	d-xs (‰)	Calcite δ^{18}O (‰)	ΔT from stalagmite-water δ^{18}O (°C)
Uemura et al. (2016)	Drip water (Modern)		0.0				-5.59 ± 0.29		-32.5 ± 2.2	12.2		
This study	GYKN-2_41.3-43.2	42.3	13.8	122.3	9957	-5.67	-4.90 ± 0.63	-28.4	-27.3 ± 3.3	17.0	-4.67	-6.7
	GYKN-2_46.0-48.1_a	47.1	14.3	107.5	6056	-5.33		-32.8		9.9	-4.06	-8.0
	GYKN-2_46.0-48.1_b	47.1	14.3	222.2	16902	-3.89		-23.6		7.5	-3.86	-1.9
	GYKN-2_53.4-55.3	54.4	14.7	134.3	20069	-4.54		-26.9		9.5	-3.80	-5.4
	GYKN-2_72.5-74.4_b	73.5	15.2	62.1	6641	-5.05		-27.4		13.0	-3.77	-8.1
	GYKN-2_72.5-74.4_c	73.5	15.2	95.3	24636	-4.88		-24.6		14.5	-3.31	-9.4
	GYKN-2_165.3-167.2_a	166.3	22.4	174.9	15772	-4.50	-4.91 ± 0.28	-23.12	-26.1 ± 2.9	12.8	-4.18	-3.3
	GYKN-2_193.6-197.6	195.6	23.2	239.9	12661	-5.03		-28.71		11.5	-4.45	-4.6
	GYKN-2_214.2-216.0	215.1	23.3	116.1	19927	-5.01		-28.49		11.6	-3.83	-7.5
	GYKN-2_224.0-226.2	225.1	23.4	117.8	13707	-5.10		-24.17		16.6	-4.00	-7.2

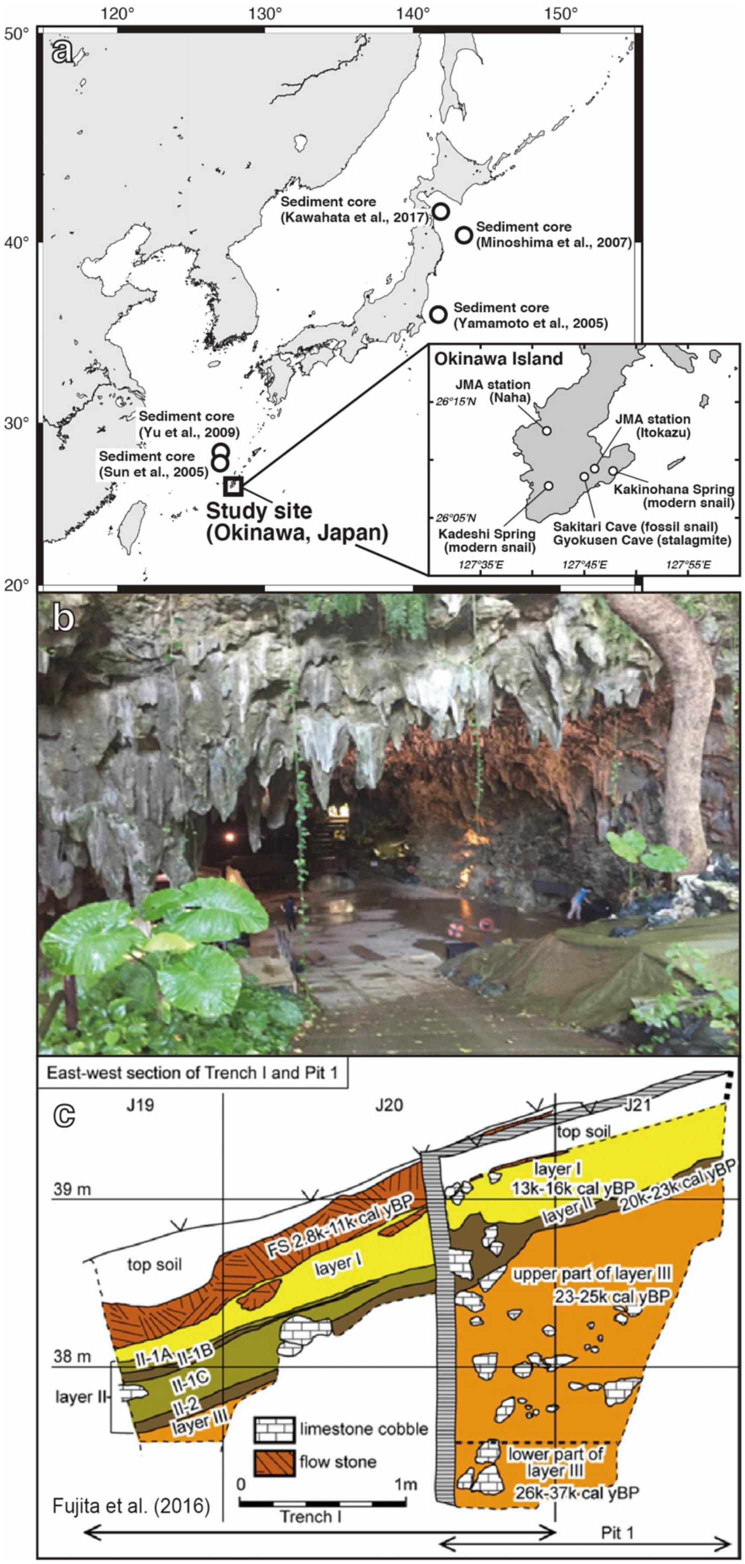

Figure S1. (a) Map of eastern Asia and the northwestern Pacific region derived from General Mapping Tool (GMT: Wessel & Smith, New, improved version of Generic Mapping Tools released, *EOS Trans. AGU*, 79 (47), pp.579 [1998]), showing the study site (26.2°N, 127.7°E) and the locations of previously published ocean sediment records. In the southern part of Okinawa Island, fossil freshwater snail samples were collected from an archaeological site in Sakitari Cave (b: photo); a stalagmite sample was collected from Gyokusen Cave in Nanjo City. Living freshwater snail samples were collected from two sites (Kakinohana Spring in Nanjo City and Kadeshi Spring in Itoman City) around the caves. Weather observation data are available from JMA meteorological stations (Naha Station for 1891–2020 and Itokazu Station for 1977–2020). (c) Fossils used in this study were taken from Layer I (16.1–13.4 ka) and Layer II-2 (23.1–22.5 ka) in the cave [24]. A previous archaeological study showed that AMS ^{14}C dates of 42 woody charcoal, seashell, snail, and crab samples are highly consistent with the stratigraphy, indicating little post-depositional disturbance. These ages suggest near continuous deposition during 36.5–13.0 ka with no erosion and hiatuses. The Pleistocene strata are well preserved beneath a Holocene flowstone (ca. 11.0–2.8 ka).

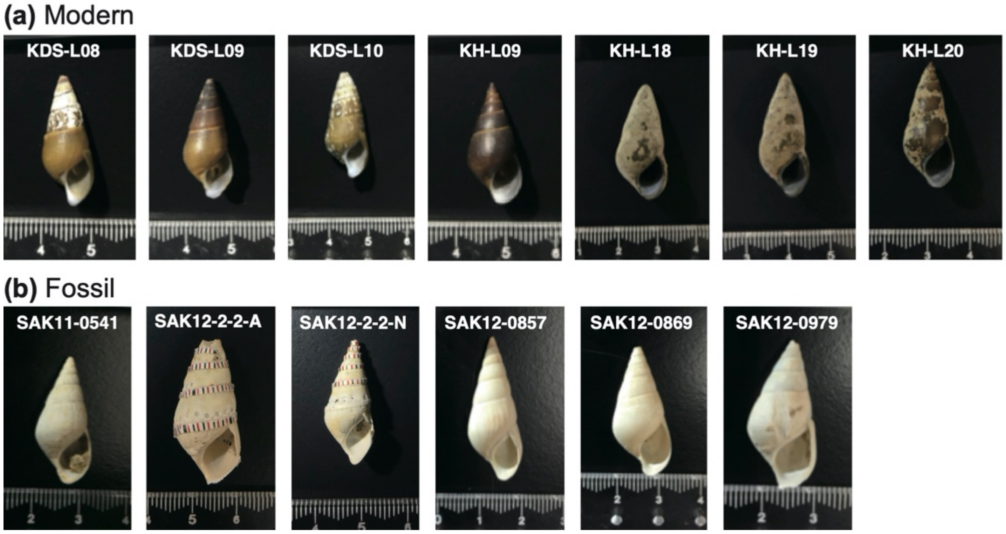

Figure S2. Photographs of **(a)** living (n = 7) and **(b)** fossil (n= 6) samples of *Semisulcospira* sp.

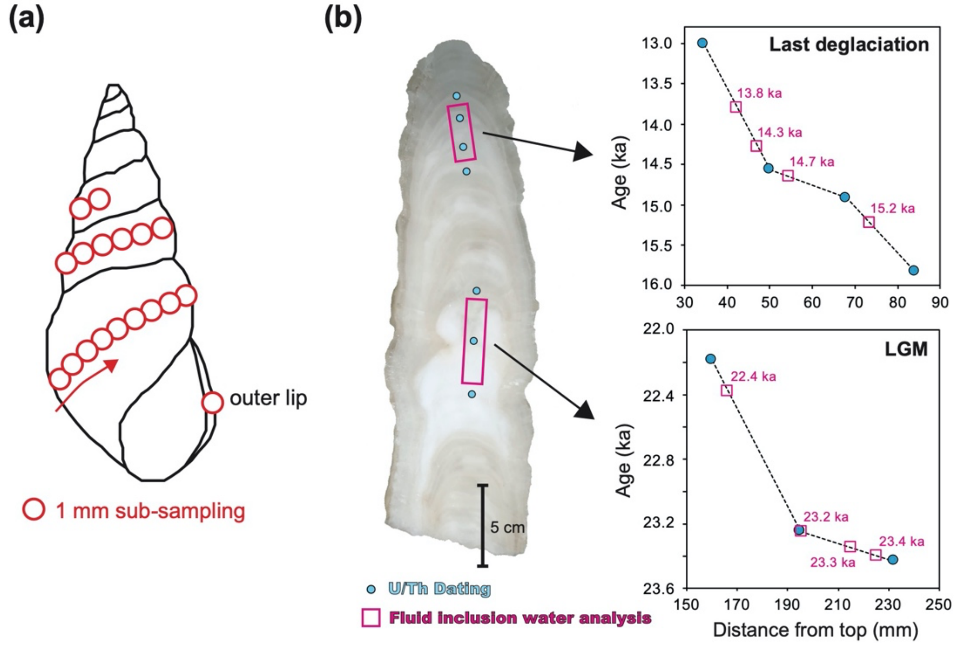

Figure S3. (a) Schematic illustration of the sub-sampling at 1 mm resolution of the snail shells for geochemical analyses. **(b)** Photograph of the stalagmite GYKN-2 showing the sub-sampling for U–Th dating (light blue) and fluid inclusion water $\delta^{18}O$ analysis (pink). The ages of the fluid inclusion waters were obtained by linear interpolation of the U–Th dating results.

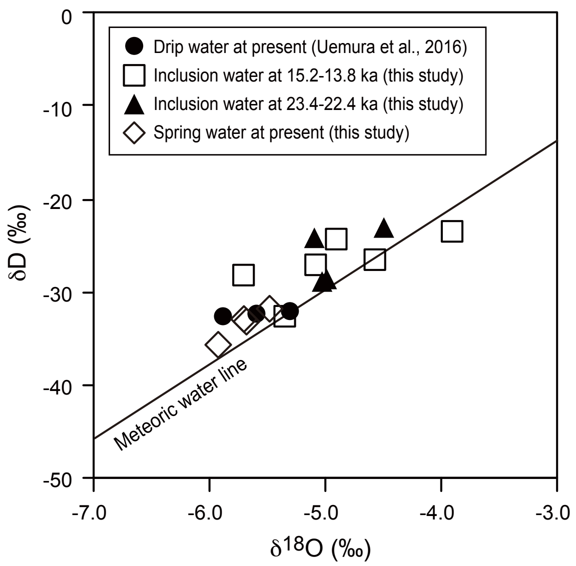

Figure S4. Plot of fluid inclusion water $\delta^{18}O$ and δD values for 15.2–13.8 ka and 23.4–22.4 ka (this study), compared with modern drip waters in Gyokusen Cave [21], modern spring waters (this study), and the meteoric water line.

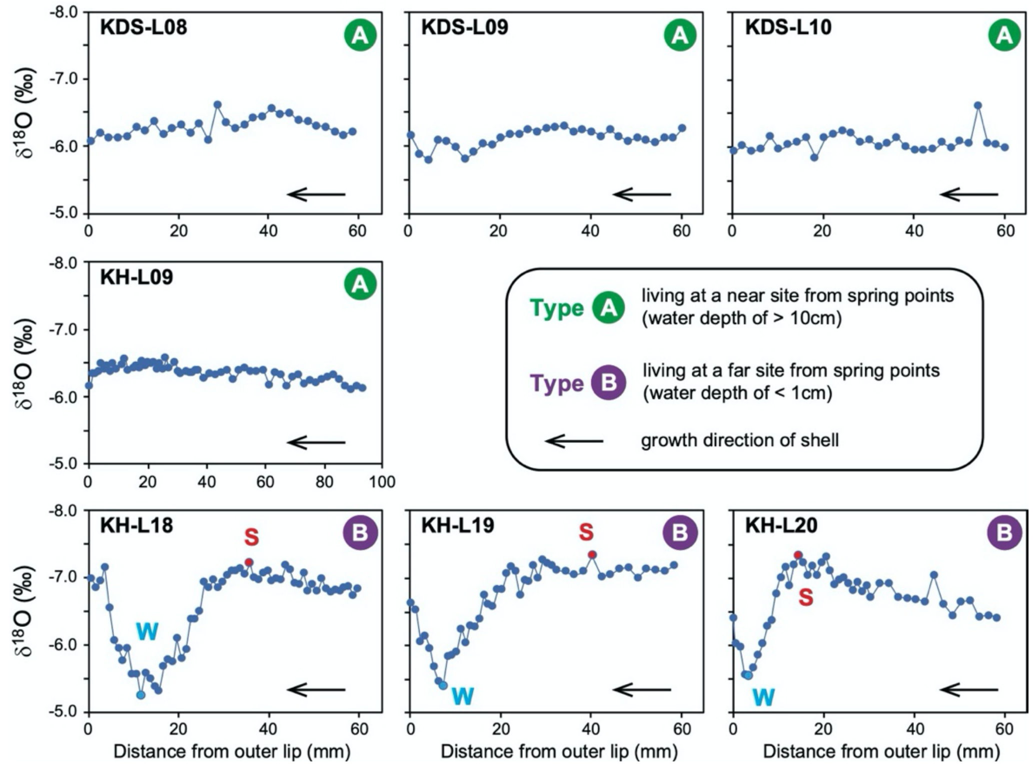

Figure S5. $\delta^{18}O$ profiles of modern freshwater snail samples (KH = Kakinohana Spring; KDS = Kadeshi Spring). The lowest and highest values in a seasonal cycle were used as the annual maximum (red circles) and minimum (blue circles) temperatures during summer and winter, respectively.
